# Dipolar Copper(I) Complexes: A Novel Appealing Class of Highly Active Second-Order NLO-Phores

**DOI:** 10.3390/molecules30051009

**Published:** 2025-02-21

**Authors:** Alessia Colombo, Claudia Dragonetti, Francesco Fagnani, Dominique Roberto, Simona Fantacci

**Affiliations:** 1 Dipartimento di Chimica, Università degli Studi di Milano, UdR INSTM di Milano, Via C. Golgi 19, I-20133 Milan, Italy; alessia.colombo@unimi.it (A.C.); claudia.dragonetti@unimi.it (C.D.); dominique.roberto@unimi.it (D.R.); 2 Computational Laboratory for Hybrid/Organic Photovoltaics (CLHYO), Istituto di Scienze e Tecnologie Chimiche “Giulio Natta” SCITEC, Consiglio Nazionale delle Ricerche (CNR), Via Elce di Sotto 8, I-06213 Perugia, Italy; simona.fantacci@cnr.it

**Keywords:** copper complexes, EFISH, nonlinear optics

## Abstract

The second-order nonlinear optical (NLO) properties of the known heteroleptic complex [Cu(1,10-phenanthroline)xantphos][PF_6_] (complex **1**) and the related new complexes [Cu(5-NO_2_-1,10-phenanthroline)xantphos][PF_6_] and [Cu(5-NO_2_-1,10-phenanthroline)(dppe)][PF_6_] (dppe = 1,2-bis(diphenylphosphino)ethane) (complexes **2** and **3**) were investigated in solution by the EFISH (Electric Field-Induced Second Harmonic generation) technique, working at a non-resonant wavelength of 1907 nm. It turned out that they are characterized by large μβ values (957–1100 × 10^−48^ esu), much higher than that of the Disperse Red One benchmark. Unexpectedly, the homoleptic complex [Cu(2-mesityl-1,10-phenanthroline)_2_][PF_6_] (complex **4**) shows a similar high second-order NLO response. Quantum chemical calculations based on Density Functional Theory (DFT) methods have been carried out to give insight into the electronic structure of the investigated complexes in relation to NLO properties. This investigation, which represents the first EFISH study on copper(I) complexes, opens a convenient route for the development of low-cost dipolar NLO-active heteroleptic [Cu(P^P)(N^N)][PF_6_] and homoleptic [Cu(N^N)_2_][PF_6_] complexes.

## 1. Introduction

Nonlinear optics (NLO) is concerned with optical phenomena, in which an electromagnetic field and a molecule or bulk material interact, giving electromagnetic fields with new frequencies, phases, or other physical features [1,2,3]. These optical characteristics depend on the compound or bulk material’s polarizability. Typically, in dipolar molecules, good quadratic hyperpolarizabilities (β) can be reached when an electron acceptor moiety (A) is connected through a polarizable bridge to an electron donor moiety (D). Non-dipolar structures, such as octupolar molecules with a central D or A core linked to three π-delocalized A or D branches, can also be characterized by good second-order NLO properties [1,2,3]. Experimentally, the quadratic hyperpolarizability of dipolar compounds can be determined in a solution by the EFISH (Electric Field-Induced Second Harmonic generation) technique, which gives the μβ product, where μ is the dipole moment in the ground state and β corresponds to the projection of the vectorial contribution of the quadratic hyperpolarizability tensor along the *μ* axis, working at a wavelength λ [4,5]. In this technique, dipolar *push–pull* molecules are aligned with the help of an electric field. Another way to determine the quadratic hyperpolarizability in a solution is the HRS (Hyper–Rayleigh Scattering, sometimes called Harmonic Light Scattering, HLS) technique, which is suitable for all compounds (dipolar or not) [6,7,8] but suffers from the restriction of the possible overestimation of values of the quadratic hyperpolarizability due to multiphoton fluorescence.

In the last two decades, there has been a lot of scientific and technological interest in the study of compounds with NLO properties as molecular building block materials for electro-optical devices, optical communications, and optical data storage and processing [9]. Among them, coordination metal complexes constitute an important class of NLO chromophores that can give more flexibility with respect to organic compounds because they are usually characterized by low-energy and high-intensity intraligand (IL), ligand-to-metal (LM), and metal-to-ligand (ML) charge transfer (CT) electronic transitions, which can be tuned by the nature, oxidation state and coordination sphere of the metal [10,11,12,13,14,15,16,17,18] and even by the number of f electrons [19]. The metal may be regarded as the donor, the acceptor, or the bridge moiety of a donor acceptor organic network [20,21]. In addition, the great diversity of the geometries of coordination compounds has introduced a new dimension to the NLO field [22].

In this context, NLO-active coordination compounds based on low-cost metals are today of particular interest for NLO applications. Many zinc [23,24,25,26] and iron [27,28,29,30,31] complexes with significant second-order NLO properties have been studied. However, surprisingly, copper complexes have been less investigated, although they proved their great potential in dye-sensitized solar cells [32,33,34,35,36,37,38,39,40,41,42,43,44,45] and in other fields, such as photocatalysis [46,47,48,49] and light-emitting electrochemical cells [50].

It has been reported that some powdered Cu(I) hybrid inorganic–organic materials and coordination copper-based polymers can be characterized by good second-order NLO properties, as determined by the Kurtz–Perry method [51,52,53,54,55,56,57,58,59]. Thus, a high NLO response was reported for a *powdered* layered material of formula [*trans*-4(4-dimethylaminostyryl)-1-methylpyridinium][Cu_5_I_6_] [51]; polymethylmetacrylate films based on this material also gave a good second harmonic generation response [60].

Some work has been reported on the second-order NLO properties in a solution of various dipolar Schiff base Cu(II) complexes [22]. Thus, the dipolar bis(salicylaldiminato)Cu(II) complex, in which the copper ion templates a noncentrosymmetric structure and acts as a donor of a donor acceptor system by means of MLCT transitions, is characterized by a significant second-order NLO response, as determined by the EFISH technique in a solution (μβ = −350 × 10^−48^ esu, working with an incident wavelength of 1.34 μm) [61,62]. The addition of suitable electron acceptor and electron donor substituents on the Schiff base ligands can lead to a higher second-order NLO response [63]. Cu(II) complexes with two differently substituted salicylaldehyde moieties are characterized by μβ in the range 108–672 × 10^−48^ esu, depending on the nature of the substituents, working with an incident wavelength of 1.907 μm [64]. A good EFISH μβ value has also been observed for a Cu(II) complex bearing an unsymmetrical Schiff base of S-methylisothiosemicarbazide (−538 × 10^−48^ esu) [65], also working with an incident wavelength of 1.907 μm chosen so its second harmonic at 954 nm occurs far from any significant absorption band of the investigated complex so that dispersive enhancements of the EFISH response are minimized [66].

**Scheme 1:**
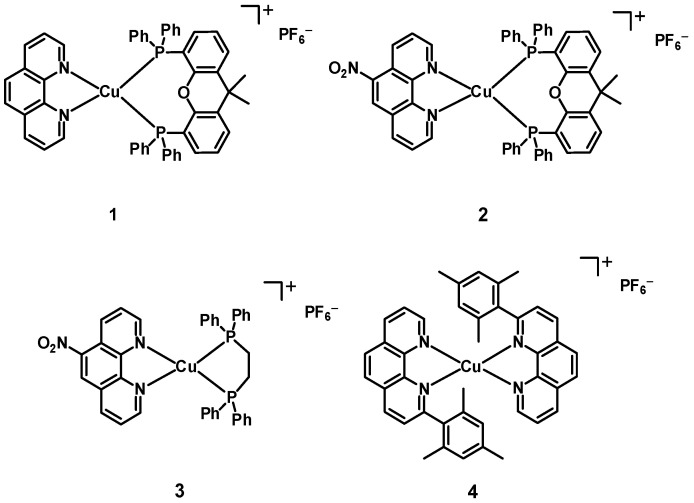
Chemical structures of the investigated Cu(I) complexes.

On the other hand, whereas it is known that non-dipolar octupolar tetrahedral bipyridyl Cu(I) complexes can be characterized by a good NLO response, as determined by the HLS technique [24,67,68,69], to our knowledge, the second-order NLO properties of a dipolar Cu(I) complex have never been reported. This observation prompted us to investigate those of complex **1**, a known photocatalyst [46], bearing a simple 1,10-phenanthroline and xantphos (Scheme 1). Curious to see the effect on the NLO properties of the introduction of an electron-withdrawing substituent on the phenanthroline, we prepared and studied the novel complex **2** and the related complex **3**, bearing a different diphosphine. For comparison, we also investigated the second-order NLO properties of complex **4**, a useful redox mediator for DSSCs [70], bearing two 1-10-phenanthrolines substituted with one mesityl group in position 2 to ensure a dipolar character [71].

## 2. Results and Discussion

### 2.1. Preparation of the Complexes

**Scheme 2:**
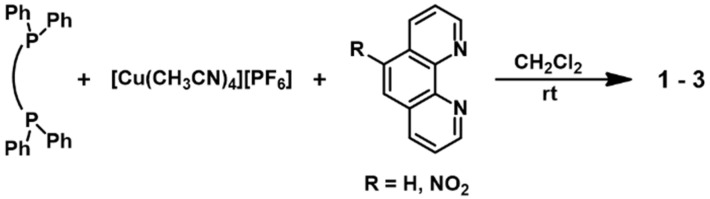
Synthesis of complexes **1**–**3**.

Complexes **1**–**3** are readily prepared by the reaction of [Cu(CH_3_CN)_4_][PF_6_] [72] with the proper diphosphine and phenanthroline in dry CH_2_Cl_2_ under argon at room temperature (Scheme 2). Precipitation from dichloromethane/diisopropylether affords the pure complexes in 82–92% yields as yellow or orange solids. Complex **4** can be prepared as previously described [70].

### 2.2. UV-Vis Absorption Spectra and Computational Modeling

**Figure 1:**
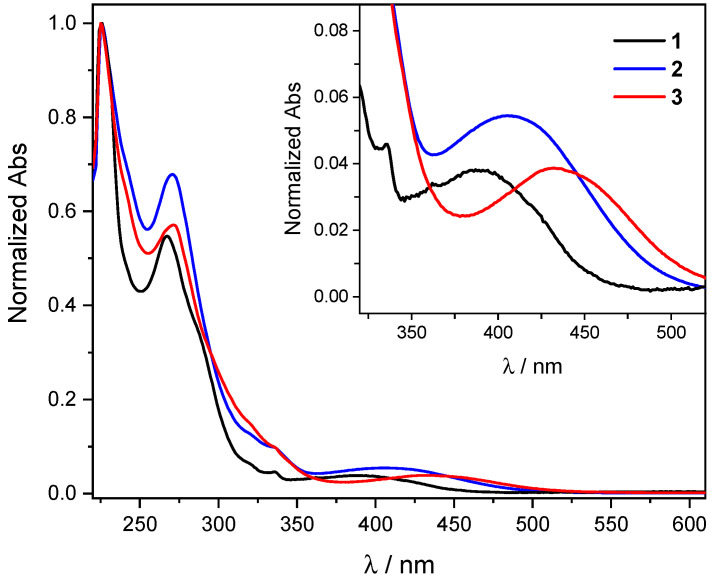
UV-Vis absorption spectra of complexes **1**–**3** in CH_2_Cl_2_. The weak bands at longer wavelengths are shown on an expanded scale for clarity.

The UV-Vis absorption spectra of complexes **1**–**3** in CH_2_Cl_2_ solution are shown in Figure 1.

As expected by analogy to previously described heteroleptic [Cu(P^P)(N^N)][PF_6_] compounds [50], complexes **1**–**3** are characterized by a low energy large absorption band centered in the range of 388–434 nm due to MLCT transitions. It has been reported that in heteroleptic four-coordinated [Cu(P^P)(N^N)]^+^ complexes (where P^P is a diphosphine and N^N is a diamine, such as a bipyridine or phenanthroline), the HOMO is largely located on the copper(I) center with some contribution from the phosphorus atoms, while the LUMO is localized on the N^N ligand, affording MLCT transitions with a high charge transfer character [50]. The replacement of the unsubstituted 1,10-phenanthroline in complex **1** by the 5-nitro-1,10 phenanthroline (complex **2**) leads to a red shift of the low-energy λ_max_ reasonably due to the lowering of the LUMO π* orbital energy of the 1,10-phenanthroline upon the introduction of the nitro group, with a consequent reduction in the HOMO-LUMO gap.

**Figure 2:**
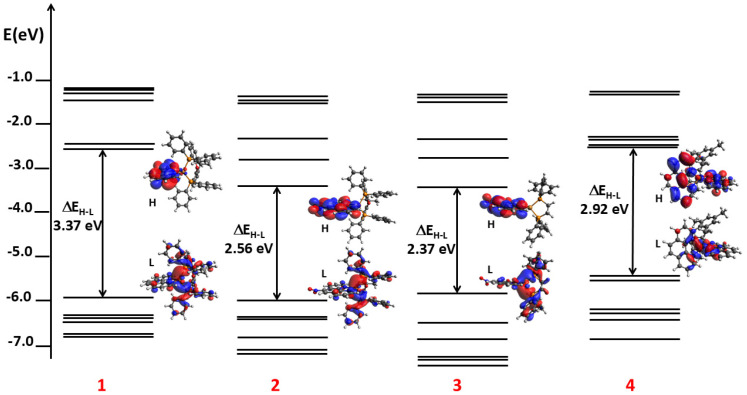
Energy levels of the Highest Occupied Molecular Orbitals (HOMOs) and Lowest Unoccupied Molecular Orbitals (LUMOs) of the four Cu(I) complexes in the −7.0–−1.0 eV range. The isodensity plot (isodensity contour = 0.02) of the HOMO and LUMO of all systems are reported.

To confirm this analysis and to provide insight into the electronic properties of the four Cu(I) complexes, the energies and electronic density plots of the frontier molecular orbitals and the relative HOMO-LUMO energy gap have been computed by means of methods based on the Density Functional Theory (DFT). They are schematized in Figure 2.

DFT calculations confirm that the introduction of the nitro group on the phenantroline ligand has a trascurable effect on the HOMO (0.06 eV) but has a strong impact on the LUMO, stabilizing it by 0.83 eV, as expected for the NO_2_ electron-withdrawing nature. As we can see in Figure 2, both the LUMOs of complexes **1** and **2** are completely delocalized in the NO_2_ phenantroline and phenantroline ligands, while both the HOMOs are delocalized on the Cu. Based on this electronic structure, the lowest transition of the relative UV-Vis spectra of complexes **1** and **2** is reasonably an MLCT transition, having as a starting state the HOMO and as an arriving state the LUMO, thus reflecting in a red shift the low-energy absorption band going from **1** to **2**. The replacement of xantphos with 1,2-bis(diphenylphosphino)ethane, going from complex **2** to complex **3**, leads to a destabilization of the HOMO (0.19 eV) and, therefore, to a further decrease in the HOMO-LUMO gap, which is in agreement with the additional red shift of the low-energy absorption band observed in the experimental UV-Vis spectra (Figure 1). The LUMO of the homoleptic complex **4** with 2-mesityl-1,10-phenanthrolines is similar to that of **1,** but its HOMO is more destabilized, leading to a lower HOMO-LUMO gap with respect to complex **1**.

### 2.3. Study of the Second-Order NLO Properties

The second-order NLO response of complexes **1**–**4** was investigated by the EFISH method [4,5], which provides direct information at a molecular level on the intrinsic dipolar second-order NLO properties. This technique affords the product μβ, where the μ term is the ground state dipole moment and the β term is the vector component of the quadratic hyperpolarizability tensor along the *μ* axis using an incident wavelength λ. A molecule characterized by an EFISH μβ value higher than that of Disperse Red One (μβ = 440 × 10^−48^ esu, working with an incident wavelength of 1907 nm) is commonly considered of interest for second-order NLO applications [73,74]; Disperse Red One (*trans*-4,4′-O_2_NC_6_H_4_N = NC_6_H_4_N(CH_2_CH_2_OH)Et) has been used in efficient electro-optic polymeric poled films and is generally taken as a reference.

It is worth pointing out that in order to avoid resonance enhancements that would lead to an overestimated value of the quadratic hyperpolarizability, it is necessary to work with an incident wavelength characterized by a second harmonic far from the absorption wavelengths of the investigated molecule [66]. In order to study complexes **1**–**4**, we used an incident wavelength of 1907 nm, obtained by Raman shifting the 1064 nm wavelength from a Q-switched mode-locked Nd:YAG laser.

**Scheme 3:**
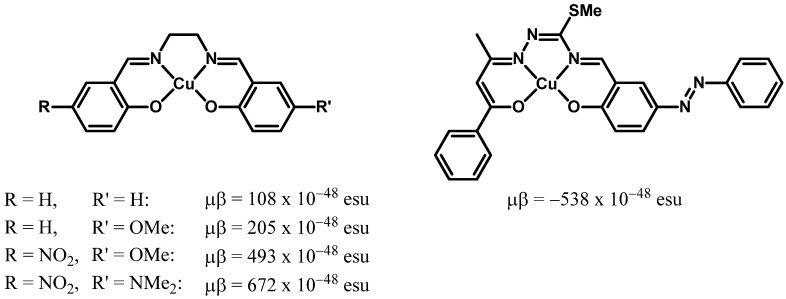
EFISH μβ values of previously reported Cu(II) complexes [64,65].

The EFISH μβ values of complexes **1**–**4**, measured as solutions in CHCl_3_, are shown in Table 1. They are in the range of 957–1100 × 10^−48^ esu, much higher than that of Disperse Red One and of that previously reported for more laborious Cu(II) complexes, determined under the same conditions with an incident wavelength of 1907 nm [64,65] (Scheme 3).

The investigated dipolar Cu(I) complexes are characterized by a positive value of μβ, which can reasonably be assigned to a positive value of Δμ_eg_ (difference of the dipole moment going from the excited to the ground state), which reflects an increase in the dipole moment upon excitation, according to the two-level model [4,75].

The remarkably high second-order NLO response in complexes **1**–**3** can be easily attributed to the MLCT transitions from the copper (diphosphine) moiety to the 1,10-phenanthroline. Indeed, the replacement of the unsubstituted 1,10-phenanthroline in complex **1** by the 5-nitro-1,10 phenanthroline (complex **2**) leads to a red shift of the low-energy λ_max_ (Table 1) and to a small increase in the μβ value. This behavior can be easily explained by similarity with previous studies on [Ir(ppy)_2_(5-R-1,10-phenanthroline)][PF_6_] (ppy = cyclometallated 2-phenylpyridine, R = H, NO_2_), in which the strong NLO response (μβ = −1270 and −2230 × 10^−48^ esu for R = H and NO_2_, respectively) is controlled by MLCT processes from the Ir (ppy)_2_ moiety, acting as a donor push system to the π* orbitals of the 1,10 phenanthroline and acting as an acceptor *pull* system [76]. Like in this class of cyclometalated Ir complexes, as confirmed by DFT calculations (Figure 2), the observed red shift of the λ_max_ going from complex **1** to complex **2** can be attributed to the lowering of the LUMO π* orbital energy of the 1,10-phenanthroline upon the introduction of the nitro group, with a consequent reduction in the HOMO-LUMO gap and a higher NLO response. In any case, the increase in the μβ value upon the introduction of the nitro group is relatively small, suggesting that there is not a strong influence of the nature of the 1,10-phenantroline substituents on the second-order NLO properties of this kind of heteroleptic dipolar [Cu(P^P)(N^N)]^+^ complex. The effect of the nature of the diphosphine also appears negligible, as observed when going from complex **2** to complex **3** by the replacement of xantphos with 1,2-bis(diphenylphosphino)ethane (Table 1).

The second-order NLO properties of homoleptic Cu(I) complexes have never been studied by the EFISH technique because the investigated complexes were, up to now, non-dipolar compounds with a D_2d_ symmetry. Thus, it has been reported that octupolar tetrahedral bipyridyl Cu(I) complexes can be characterized by good second-order NLO properties, as determined by the HLS technique [67,68,69]. In these systems, the NLO response is controlled by ILCT transitions, when the bipyridine bears electron donor substituents with the Cu(I) center acting as an electron acceptor, whereas, in the absence of donor groups on the bipyridine, the response is due to MLCT dπ(Cu)-π*(bipyridine) transitions characterized by a λ_max_ of around 450 nm [69]. We were curious to know the value of μβ of a dipolar homoleptic Cu(I) complex, such as **4**, bearing two simple 1,10-phenanthrolines in which the presence of the bulky mesityl groups protects as a kiss lock the Cu(I) center and ensures the dipolar character [71]. Remarkably, it turned out that also complex **4** is characterized by a high EFISH μβ value of 960 × 10^−48^ esu.

It is known that the concept, and, therefore, the experimental determination, of the dipole moment of charged species is quite problematic [76]; therefore, unfortunately, it is not possible to calculate the β value of complexes **1**–**4** from the product μβ obtained by the EFISH technique. In any case, from an application point of view, it is important to maximize μβ. Clearly, all the investigated heteroleptic and homoleptic Cu(I) complexes are of interest for NLO applications, having a similar large μβ value that is more than twice that of Disperse Red One, which has been used in efficient electro-optic polymeric poled films [73].

It is worth pointing out that the investigated complexes are both easily prepared and very stable. Moreover, the low cost of copper, a truly green circular material with an infinite life cycle [77], makes these second-order NLO-active Cu(I) complexes particularly appealing for the development of sustainable and environmentally friendly telecommunications, computing, optoelectronic devices, and optical communication.

## 3. Materials and Methods

Solvents and reagents were bought from Sigma-Aldrich (St. Louis, MO, USA) and used as received. Complexes [Cu(CH_3_CN)_4_][PF_6_] [72] and **4** [70] were synthesized according to the literature.

NMR spectra were recorded on a Bruker AV III 400 MHz spectrometer (Berlin, Germany). ^1^H chemical shifts are reported in parts per million (ppm), whilst the coupling constants (J) are expressed in Hz. The abbreviations reported in brackets refer to the multiplicities, where s, d, t, q, and m indicate, respectively, singlet, doublet, triplet, quartet, and multiplet.

Electronic absorption spectra in a solution were obtained with a UV-3600i Plus UV-VIS-NIR spectrophotometer (Shimadzu Italia S.r.l., Milan, Italy).

Elemental analysis was carried out with a PerkinElmer CHN 2400 (Waltham, MA, USA) instrument in the Analytical Laboratories of the Department of Chemistry at the University of Milan.

### 3.1. Procedure for the Synthesis of Complexes ***1***–***3***

A solution of the proper diphosphine (0.110 mmol) in dry CH_2_Cl_2_ (10 mL) was added under vigorous stirring to a solution of [Cu(CH_3_CN)_4_][PF_6_] (0.100 mmol) in dry CH_2_Cl_2_ (10 mL) under argon atmosphere; after 1 h, a solution of the proper phenanthroline (0.110 mmol) in dry CH_2_Cl_2_ (3.0 mL) was added dropwise, and the reaction mixture was left under argon atmosphere for 1 h. The solution was then concentrated to half of its volume under reduced pressure, and *i*Pr_2_O was added to precipitate the product, which appeared as a yellow or orange solid.

Complex **1**: pale yellow solid, 51 mg, yield = 90%.

^1^H-NMR (400 MHz, CD_2_Cl_2_): δ 8.50 (d, *J* = 6.3 Hz, 4H), 8.05 (s, 2H), 7.75 (d, *J* = 7.8 Hz, 2H), 7.69 (t, *J* = 6.3 Hz, 2H), 7.25 (t, *J* = 7.2 Hz, 4H), 7.20 (t, *J* = 7.7 Hz, 2H), 7.06 (t, *J* = 7.4 Hz, 8H), 6.94 (m, 8H), 6.65 (m, 2H), 1.82 (s, 6H).

UV-Vis (CH_2_Cl_2_): λ_max_ (ε/10^3^ M^−1^ cm^−1^) = 227 nm (105.0), 268 nm (63.6), 388 nm (3.4).

Elemental analysis: calcd. for C_51_H_40_CuF_6_N_2_OP_3_: C, 63.32; H, 4.17; N, 2.90; found: C, 64.10; H, 4.19; N, 2.99.

Complex **2**: yellow solid, 63 mg, yield = 82%.

^1^H-NMR (400 MHz, CD_2_Cl_2_): δ 9.17 (d, *J* = 8.0 Hz, 1H), 8.88 (s, 1H), 8.67 (m, 3H), 7.87 (dd, *J*_1_ = 8.0 Hz, *J*_2_ = 4.2 Hz, 2H), 7.77 (d, *J* = 8.0 Hz, 2H), 7.25 (m, 6H), 7.10 (dd, *J*_1_ = 12.2 Hz, *J*_2_ = 8.0 Hz, 8H), 6.96 (m, 8H), 6.73 (m, 2H), 1.81 (s, 6H).

UV-Vis (CH_2_Cl_2_): λ_max_ (ε/10^3^ M^−1^ cm^−1^) = 226 nm (147.8), 271 nm (92.7), 405 nm (7.0).

Elemental analysis: calcd. for C_51_H_39_CuF_6_N_3_O_3_P_3_: C, 60.51; H, 3.88; N, 4.15; found: C, 59.30; H, 3.90; N, 4.03.

Complex **3**: pale orange solid, 74 mg, yield = 92%.

^1^H-NMR (400 MHz, CD_2_Cl_2_): δ 9.36 (d, *J* = 8.1 Hz, 1H), 9.07 (s, 1H), 8.97 (d, *J* = 8.1 Hz, 1H), 8.86 (m, 2H), 8.07 (m, 2H), 7.48 (m, 3H), 7.41 (m, 13H), 7.22 (m, 4H), 2.80 (t, *J* = 5.8 Hz, 4H).

UV-Vis (CH_2_Cl_2_): λ_max_ (ε/10^3^ M^−1^ cm^−1^) = 226 nm (95.5), 271 nm (56.5), 434 nm (4.6).

Elemental analysis: calcd. for C_38_H_31_CuF_6_N_3_O_2_P_3_: C, 54.85; H, 3.75; N, 5.05; found: C, 55.01; H, 3.71; N, 5.12.

### 3.2. Computational Detail

DFT calculations were performed on the **1**–**4** Cu(I) complexes using the quantum chemistry program package Gaussia09 (G09) [78]. The molecular geometry of the four complexes has been optimized by means of the B3LYP exchange correlation functional [79], 6–311 g **, the basis set [80,81] for all atoms, including the CH_2_Cl_2_ effects, by the conductor-like polarizable continuum model (CPCM) as implemented in G09 [82,83,84,85]. The computed electronic structure in terms of energy and nature of the frontier orbitals was analyzed.

### 3.3. EFISH Measurements

All copper complexes were studied by the EFISH technique [4,5] in CHCl_3_ solutions with a 10^−3^ M concentration using a non-resonant incident wavelength of 1907 nm obtained by Raman shifting the fundamental 1064 nm wavelength produced by a Q-switched mode-locked Nd^3+^:YAG laser manufactured by Atalaser. The apparatus used for EFISH measurements is a prototype made by SOPRA (Bois-Colombes, France). The laser pulses focused onto a liquid cell and synchronized with a dc field applied to a solution containing the complex. By translation of the cell perpendicularly to the incident beam, the variation of the propagation length in the solution creates a Maker fringe whose amplitude and periodicity are related to the nonlinearity of the solution. The reported values of μβ are the mean values of 16 measurements performed on the same sample. The sign of µβ is determined by comparison with the reference solvent (CHCl_3_). EFISH values are obtained according to the “phenomenological convention” [86]. Further details of the experimental methodology and data analysis are reported elsewhere [5].

## 4. Conclusions

In conclusion, this work shows evidence for the first time that simple dipolar Cu(I) complexes can be characterized by excellent μβ values, much larger than that of the benchmark Disperse Red One and of previously reported Cu(II) complexes. Remarkably, their NLO response is approaching the best values obtained for coordination compounds based on much more expensive metals, such as iridium and platinum, an interesting aspect from an economical point of view taking into account the abundance and low cost of copper. These results open the route for the development of both dipolar NLO-active heteroleptic [Cu(P^P)(N^N)]^+^ and homoleptic [Cu(N^N)_2_]^+^ complexes, which are of particular interest for the development of sustainable telecommunications, computing, optoelectronic devices, and optical communication. As a future research direction, it would be of particular interest to use them for the fabrication of electro-optic modulators [9] since optical signal modulation plays a key role in today’s communication technology.

## Figures and Tables

**Table 1:** Second-order NLO properties of the investigated Cu(I) complexes.

Complex	λ_max,abs/_nm [ε/10^3^ M^−1^ cm^−1^]	μβ/10^−48^ esu ^a^
**1**	388 [3.4] ^b^	995
**2**	405 [7.0] ^b^	1100
**3**	434 [4.6] ^b^	957
**4**	451 [3.6] ^c^	960

^a^ In CHCl_3_ at 10^−3^ M, the experimental error is ± 10%; ^b^ in CH_2_Cl_2_; ^c^ in CH_3_CN.

## Data Availability

All data are already included in the manuscript.
